# Associations between Film Preferences and Risk Factors for Suicide: An Online Survey

**DOI:** 10.1371/journal.pone.0102293

**Published:** 2014-07-16

**Authors:** Benedikt Till, Ulrich S. Tran, Martin Voracek, Gernot Sonneck, Thomas Niederkrotenthaler

**Affiliations:** 1 Suicide Research Unit, Institute of Social Medicine, Center for Public Health, Medical University of Vienna, Vienna, Austria; 2 Department of Basic Psychological Research and Research Methods, School of Psychology, University of Vienna, Vienna, Austria; 3 Georg Elias Müller Department of Psychology, Georg August University of Göttingen, Göttingen, Germany; 4 Crisis Intervention Center and Ludwig Boltzmann Institute for Social Psychiatry, Vienna, Austria; Cognitive Brain Research Unit, Finland

## Abstract

Several studies indicate that exposure to suicide in movies is linked to subsequent imitative suicidal behavior, so-called copycat suicides, but little is currently known about whether the link between exposure to suicidal movies and suicidality is reflected in individual film preferences. 943 individuals participated in an online survey. We assessed associations between preferred film genres as well as individual exposure to and rating of 50 pre-selected films (including 25 featuring a suicide) with suicidal ideation, hopelessness, depression, life satisfaction, and psychoticism. Multiple regression analyses showed that preferences for film noir movies and milieu dramas were associated with higher scores on suicidal ideation, depression and psychoticism, and low scores on life satisfaction. Furthermore, preferences for thrillers and horror movies as well as preferences for tragicomedies, tragedies and melodramas were associated with higher scores of some of the suicide risk factors. There was also a dose-response relationship between positive rating of suicide films and higher life satisfaction. Due to the cross-sectional design of the study causality cannot be assessed. Individual film genre preferences seem to reflect risk factors of suicide, with film genres focusing on sad contents being preferred by individuals with higher scores on suicide risk factors. However, suicide movies are more enjoyed by viewers with higher life satisfaction, which may reflect a better ability to cope with such content.

## Introduction

Watching movies is the number one leisure activity in Western societies; people spend more time on watching films than on any other activity [1]–[3]. The functions of movies in a society go far beyond individual entertainment: With their constant focus on the depiction of societal norms and of ways how to deal with such norms, movies both reflect and help shape norms that are relevant in everyday life [4]. It has been shown that movies and television programs can subtly influence peoples' perception of reality, which is known as cultivation [5], [6]. Suicide is a theme very often used in Hollywood motion pictures. Stack and Bowman [3] identified 1,377 American films that contain one or more suicides between 1900 and 2009. Research on the impact of fictional media stories has shown that suicide portrayals in movies have the potential to influence suicidal behavior [7]–[9]. It is, however, unclear, whether risk factors for suicide are associated with film preferences. This assumption seems plausible, because mood management theory [10] suggests that individuals choose media inputs that modify and regulate affective experiences and mood states in desirable ways. Recipients may strive for more positive and pleasurable moods or seek to maintain their current emotional state.

It is also unclear, how risk factors for suicide are associated with film ratings. According to social comparison theory [11], humans tend to evaluate themselves by comparing their opinions and abilities to those of other people. Based on an earlier study, only individuals with lower suicide risk seem to be able to distance themselves from portrayed suicidal contents [12] and are less inclined to enjoy the movie, as reflected in individual film ratings. Earlier studies showed that particularly individuals in adverse life circumstances, who are thinking about suicide, may be most likely to seek and choose suicide-related media content and may in some cases subsequently act out their suicidal impulses [13], [14]. In accordance with this assumption, a recent study indicated that the greater the suicidal tendencies of viewers of a suicide film are, the more they tended to use the film for getting ideas about how to go through life and deal with the own problems [15].

Aside from the vast amount of literature analyzing the potential impact of media portrayals on subsequent suicides, research about associations of personal film preferences with risk factors for suicide such as suicidal thoughts or depression scores are relatively scarce. To date, it is still unclear whether preferences for suicide films or dramas in general are reflected in risk factors for suicide, and to our knowledge there is no study available that differentiates between exposure to suicide films and the individual rating of such films. Martin [16] found higher depression scores and more suicide attempts among adolescents claiming more than two exposures to suicide on television compared to individuals with fewer exposures. In a recent study, Stack et al. [17] found an association between cumulative exposure to suicide movies and suicide attempts. All these previous studies were based on small sample sizes and did not allow for the controlling for important variables such as individual film ratings or overall film consumption.

In the present study, we analyzed the associations between cumulative exposure to suicide in movies as well as the individual rating of such films with risk factors for suicidal behavior, i.e. suicidal ideation, hopelessness, depression, life satisfaction, and psychoticism, in a large sample of internet users. Furthermore, we investigated the associations between these suicide risk factors and preferences for certain film genres.

## Methods

### Participants

We conducted an online survey from December 9^th^ 2011 to August 3^rd^ 2012. Participants were recruited with posters, flyers, e-mails and public announcements at the University of Vienna and the Medical University of Vienna, Austria. In total, 2,221 individuals accessed the survey website, and 943 (42.4%) completed the online survey. This number included 631 women (66.9%) and 312 men (33.1%). Mean age was 29.7 years (*SD* = 9.7). The average level of education was high school graduation for both men (*n* = 175, 56.1%) and women (*n* = 353, 55.9%). Compared to the general population, college (sample: 39.6%; population: 13.8%) and high school graduates (sample: 56.0%; population: 14.4%) were over-represented in our sample, while individuals with an education level below high school graduation (sample: 4.4%; population: 71.6%) were under-represented [18]. Suicidal individuals were defined as participants scoring 69 points or higher on the German version [19] of the Beck Hopelessness Scale [20]. In our sample, 100 participants (10.6%) were classified as suicidal (*M* = 79.5, *SD* = 8.9) and 843 individuals (89.4%) were categorized as non-suicidal (*M* = 45.8, *SD* = 9.9).

### Ethics Statement

The study was approved by the Ethics-Committee of the Medical University of Vienna and the Vienna General Hospital AKH (study protocol 1290/2012). The use of consent forms was not deemed necessary, and this approach was approved by the Ethics-Committee.

### Measures

#### Cumulative exposure to suicide in movies

To explore the relationship between cumulative viewing of suicide movies and suicidality we applied the Beach method, a technique for assessing cumulative media exposure, found in smoking and related studies of cinematic influences on behavior [4], [21]–[23], that has been used in previous studies to assess associations between exposure to suicide movies and suicide attempts [17]. Following the Beach methodology we asked participants to indicate which films they had seen from a unique list of 50 movies. The list consisted of 50 popular contemporary movies released between 1989 and 2008, with 25 movies featuring the suicide of one of the main characters. For each suicide film the list contained another movie that was released in the same year, drew a similar amount of viewers/ticket sales, and catered to the same genre, but did not feature the suicide of a protagonist. Because data on ticket sales were not available for movies that were released prior to 2004 in Austria, German data, which is considered to be comparable to Austria, were consulted for films before 2004. See Table 1 for an overview of the movies included in the list. We used two measures to assess exposure to suicide in movies: The total amount of suicide films an individual had seen and the number of viewed suicide films in relation to the number of viewed films overall.

**Table 1:** Name and Release Year of All Movies Included in the List to Assess Cumulative Exposure to Suicide in Movies.

Movies with a main character's suicide	Movies without a main character's suicide
About the Looking for and the Finding of Love (2005)	A History of Violence (2005)
Alien 3 (1992)	Cape Fear (1991)
Dead Poets Society (1989)	The War of the Roses (1989)
Donnie Darko (2001)	Ghosts of Mars (2001)
End of Days (1999)	Blair Witch Project (1999)
Fallen (1998)	Dark City (1998)
Farewell My Concubine (1993)	Benny & Joon (1993)
His Brother (2003)	Agnes and His Brothers (2004)
Leaving Las Vegas (1995)	Dead Man Walking (1995)
Love in Thoughts (2004)	The Human Stain (2003)
O (2001)	Frailty (2001)
Old Boy (2003)	Walking Tall (2004)
Open Water (2003)	Dogville (2003)
Point Break (1991)	The Hard Way (1991)
Romeo + Juliet (1996)	The Harmonists (1997)
Rossini (1997)	Knockin' on Heaven's Door (1997)
Seven Pounds (2008)	Body of Lies (2008)
Terminator 2: Judgment Day (1991)	Robin Hood: Prince of Thieves (1991)
The Eight Day (1996)	Moonlight and Valentino (1995)
The Hours (2002)	Chicago (2002)
The Lives of Others (2006)	The Constant Gardener (2005)
The Sea Inside (2004)	Crash (2004)
Thelma & Louise (1991)	Awakenings (1990)
Vanilla Sky (2001)	Unfaithful (2002)
Wanted (1999)	She's All That (1999)

#### Suicide film ratings

The participants rated the films they had seen on a 5-point-scale ranging from 0 =  *I don't like at all* to 4 =  *I like very much*. For each participant, the ratings of the suicide films were added together and divided by the number of known suicide films.

#### Preferred film genres

The participants' preferred film genres were assessed by providing a list of 22 film genres and asking them to indicate which genres they usually watch (*yes* = 1, *no* = 0). We also asked the participants to name additional film genres, when one of their preferred genres was not included. The list included the following film genres: Film noir, Milieu drama, War, Comedy, Fantasy, Sci-Fi, Martial-Arts, Action, Mystery, Adventure, Western, History, Heimatfilm, Disaster, Erotic, Tragicomedy, Tragedy, Melodrama, Romance, Thriller, Horror, and Crime. See Gehring [24] for a detailed description of the more common film genres. One of the rare genres included is *Film noir*. This is a genre that includes crime dramas that typically display a pessimistic worldview and contain cynicism or black humor (e.g., *The Third Man*, 1949, *Strangers on a Train*, 1951). It originated in the U.S. and was especially popular in the 1940s and 1950s [25]. *Heimatfilm* is another rare and culture-specific film genre that represents vintage sentimental Austrian, German and Swiss films with regional backgrounds (e.g., Sissi, 1955). *Milieu dramas* are more frequent and constitute drama films that focus on a specific social milieu and its characteristics and problems (e.g., *Slumdog Millionaire*, 2008, *Vera Drake*, 2004). The *Tragedy* genre represents drama films focusing on a character's downfall caused e.g. by a character flaw or by a major error in judgment (e.g., *The Last Samurai*, 2003, *Brokeback Mountain*, 2005) [26]. *Tragicomedies* contain elements of both, comedies and tragedies (e.g., American Beauty, 1999, Knockin' on Heaven's Door, 1997). *Melodramas* are drama films characterized by the use of plots that often deal with crises of human emotion [27] and that focus on emotional conflicts of the protagonist and his or her downfall, normally due to a string of unfortunate life events (e.g., *Gone with the Wind*, 1939, *Romeo + Juliet*, 1996) [26].

#### Suicidal ideation

The Suicide Probability Scale [28] is a 36-item self-report measure that assesses suicidal ideation. Items (e.g., “I feel people would be better off if I were dead”) are rated on a 4-point scale ranging from *None or a little of time* to *Most or all at the time*. Answers were scored from 0 to 5 points, in accordance with the manual of the questionnaire.

#### Depression

The Erlanger Depression Scale [29] uses eight self-report items (e.g. “I want to cry”) plus 1 warm-up item to assess symptoms of depression. Items are rated on a scale from 0 =  *completely wrong* to 4 =  *exactly right*.

#### Life satisfaction

The German version of the Satisfaction with Life Scale [30] is a 5-item self-report measure to assess life satisfaction (e.g. “I am satisfied with my life”). Items are rated on a scale of 1 =  *strongly disagree* to 7 =  *strongly agree*.

#### Psychoticism

The psychoticism subscale of the German short version of the Eysenck Personality Questionnaire-revised (EPS) [31] is a 14-item self-report measure that generates a score on the participants' psychoticism (e.g. “Would you take drugs which may have strange or dangerous effects?”). Items are rated on a dichotomous scale: *yes* = 1, *no* = 0.

#### Classification into suicidal and non-suicidal individuals

The German version [19] of the Beck Hopelessness Scale (BHS) [20] is a 20-item self-report measure that assesses hopelessness. Items (e.g., “My future seems dark to me“) are rated on a scale from 1 =  *very false* to 6 =  *very true*. This scale is considered to be a robust predictor of suicidal behavior and can be used to differentiate between suicidal and non-suicidal individuals [32], [33]. Based on the standard cut-off score of ≥9, which corresponds to a score of 69 in the German version [19], study participants were classified into suicidal (*n* = 100) and non-suicidal (*n* = 843) individuals [33].

### Data analysis

Preference ratings on film genres were factor-analyzed to investigate their latent structure and obtain a smaller number of composite measures of film genre preferences. Mplus 8.11 was used, performing rotated (Geomin) exploratory factor analysis using the weighted least squares means and variance adjusted (WLSMV) estimation, to assign the film genres preferences to comprehensive factors. WLSMV estimation is specifically suited to the dichotomous scoring format of film genre preference as utilized in this study. Model fit was assessed with the comparative fit index (CFI), the Tucker-Lewis index (TLI), and the root mean square error of approximation (RMSEA), using benchmarks of Hu and Bentler [34] (CFI and TLI: good fit: ≥.95, acceptable fit: ≥.90; RMSEA; good fit: <.06, acceptable fit: <.08). Since individual definitions of film genres partially overlap, we allowed inter-correlations between the factors. Four multiple linear regression analyses using forward inclusion were applied to calculate the associations between suicide risk factors and film preferences, with suicidal ideation, depression, life satisfaction, and psychoticism as respective dependent variable and the film preferences as explanatory variables. Bivariate Pearson correlations were calculated to identify potential mediators and moderators. Multivariate binary logistic regression analysis with forward inclusion was used to assess the associations between film preferences and hopelessness. The outcome variable was suicidality (suicidal vs. non-suicidal) as indicated by the BHS [20]. All regression analyses controlled for the participants' sex, age, and education were conducted with standardized scores.

## Results

For film genre preferences, five factors were extracted based on adequate model fit indices (χ^2^(131) = 277.451, *p*<.001, CFI = .963, TLI = .935, RMSEA = 0.034, 90% CI = .029 to .040) and labeled based on the highest loadings: Factor 1 loaded high on film noir movies and milieu dramas, factor 2 had high loadings on fantasy and science fiction films, factor 3 is represented strongest by adventure movies and westerns, factor 4 loaded high on tragicomedies, tragedies and melodramas, and factor 5 had high loadings on thrillers and horror movies. See Table 2 for a detailed overview of the five factors and their loadings and Table 3 for an overview of the means and standard deviations of the suicide predictors and the film preferences. High scores indicate a higher manifestation of the respective variable.

**Table 2:** Factor Loadings of Film Genre Preferences (Factors I–V) of Study Participants (n = 943).

	Film genre preferences				
Film genre	Factor I	Factor II	Factor III	Factor IV	Factor V
Film noir	**.70** ^***^	−.09	.03	.17	.03
Milieu drama	**.52** ^***^	−.30^***^	.02	.35	.00
War	**.45** ^***^	−05	.41^***^	.04	.06
Comedy	**−.42** ^***^	.05	.38^***^	.37*	.05
Fantasy	.03	**.81** ^***^	.04	.08	−.10
Sci-Fi	.33^***^	**.73** ^***^	−.01	−.05	.08
Martial arts	.24^**^	**.47** ^***^	.15	.03	−.06
Action	−.07	**.47** ^***^	.37^***^	−.17	.23^**^
Mystery	.01	**.46** ^***^	.00	.21^**^	.40^***^
Adventure	−.05	.44^***^	**.55** ^***^	−.04	.03
Western	.48^***^	.06	**.52** ^***^	−.13	.00
History	.35^**^	−.04	**.48** ^***^	.05	−.20*
Heimatfilm	.05	−.05	**.44** ^***^	.01	−.37^**^
Disaster	.08	.20*	**.43** ^***^	.12	.23^**^
Erotic	.23	.12	.27*	.21*	−.13
Tragicomedy	−.07	−.02	−.01	**.84** ^***^	.05
Tragedy	.14	.06	−.03	**.76** ^***^	.05
Melodrama	.05	.01	−.16*	**.76** ^***^	.04
Romance	−.46^**^	−.05	.27^**^	**.65** ^***^	−.14
Thriller	.04	−.02	.14	.02	**.82** ^***^
Horror	.09	.21*	−.02	.02	**.50** ^***^
Crime	.01	−.33^***^	.44^***^	.01	**.45** ^***^

*Note*. Factor loadings of film genres as estimated with Mplus. Film genres are sorted with regard to their highest factor loading (printed boldface) and in descending order.

^*^ *p*<.05, ^**^ *p*<.01, ^***^ *p*<.001 (two-tailed).

**Table 3:** Descriptive Statistics for Suicide Risk Factors and Film Preferences of Study Participants (n = 943).

Suicide Risk Factors	α	*M*	*SD*	Range
Suicidal Ideation	.89	48.52	14.12	30–140
Depression	.81	6.76	5.29	0–32
Life Satisfaction	.85	25.36	5.79	5–35
Psychoticism	.56	3.07	1.96	0–14

Film preferences	*M*	*SD*	Range
Total amount of known suicide films	7.40	4.41	0–25
Number of known suicide films per film overall	0.54	0.14	0–1
Suicide film rating	2.43	0.67	0–4
Film genre preference 1 (Film noir, Milieu drama)	0.09	0.70	−3–3
Film genre preference 2 (Fantasy, Sci-Fi)	0.03	0.74	−3–3
Film genre preference 3 (Adventure, Western)	0.04	0.72	−3–3
Film genre preference 4 (Tragicomedy, Tragedy, Melodrama)	0.04	0.75	−3–3
Film genre preference 5 (Thriller, Horror)	0.01	0.70	−3–3

*Note*. Values are means (*M*), standard deviations (*SD*), and Cronbach's alphas (α) estimated with SPSS as well as the highest and lowest possible value of the variable (Range).

Film genre preference factors 1 and 2 were significant predictors for suicidal ideation (*F*(14, 899) = 2.77, *p*<.001, Adj. *R^2^* = .03). The more the participants had preferences for film noir movies and milieu dramas, the greater was their suicidal ideation. Depression was significantly associated with preferences for film noir movies and milieu dramas as well as for tragicomedies, tragedies and melodramas (*F*(14, 899) = 3.60, *p*<.001, Adj. *R^2^* = .04). The more the participants liked drama films, the greater were their depression scores.

Preferences for film noir movies and milieu dramas and positive rating of suicide films were significant predictors for life satisfaction (*F*(14, 899) = 3.20, *p*<.001, Adj. *R^2^* = .03). The more the participants preferred film noir movies and milieu dramas, the lower was their life satisfaction. The more they liked the suicide films, the better was their life satisfaction. Psychoticism was significantly predicted by the film genre preference factors 1 and 3 (*F*(14, 899) = 5.67, *p*<.001, Adj. *R^2^* = .07). The more the participants had preferences for film noir movies and milieu dramas, the greater was their psychoticism. The more they liked adventure movies and westerns, the lower were their psychoticism scores.

Overall, young age and low education were significantly associated with higher scores on most suicide risk factors. Table 4 provides a matrix of bivariate correlations between all variables included in the multiple linear regression analyses. See Table 5 for the standardized regression coefficients and standard errors of the significant predictors and control variables of the multiple linear regression analyses. Multivariate binary logistic regression analysis with the scores on the BHS [20] to differentiate between suicidal and non-suicidal individuals as the outcome variable revealed that suicidal participants had significantly higher preferences for thrillers and horror movies (β = .33, *SE* = 0.14, *p*<.05, CI = 1.064 to 1.825).

**Table 4:** Correlations Matrix: Pearson Correlations between Risk Factors for Suicide, Film Preferences, and Socio-Demographics (n = 943).

	X1	X2	X3	X4	Y1	Y2	Y3	Y4	Y5	Y6	Y7	Y8	Z1	Z2	Z3
**Suicidal ideation (X1)**	1.00														
**Depression (X2)**	.77^***^	1.00													
**Life satisfaction (X3)**	−.62^***^	−.60^***^	1.00												
**Psychoticism (X4)**	.13^***^	.06	−.10^**^	1.00											
**N of suicide films (Y1)**	.01	−.01	−.05	.08*	1.00										
**% of suicide films (Y2)**	−.01	−.02	.00	−.01	.13^***^	1.00									
**Suicide film rating (Y3)**	.00	.03	.09^**^	.05	−.02	−.05	1.00								
**Genre: Film noir/Milieu drama (Y4)**	.15^***^	.07*	−.14^***^	.20^***^	.30^***^	.04	.08*	1.00							
**Genre: Fantasy/Sci-Fi (Y5)**	−.02	−.09^**^	.01	−03	.19^***^	.05	.03	.19^***^	1.00						
**Genre: Adventure/Western (Y6)**	−01	−.02	−.03	−.04	.14^***^	−.03	.07*	.28^***^	.49^***^	1.00					
**Genre: Tragicomedy/Tragedy/Melodrama (Y7)**	.09^**^	.14^***^	−.04	.06	.23^***^	−.02	.10^**^	.31^***^	−.14^***^	.26^***^	1.00				
**Genre: Thriller/Horror (Y8)**	.03	−.05	−.03	.02	.34^***^	.06	.03	.32^***^	.49^***^	.30^***^	.14^***^	1.00			
**Sex (Z1)** 1	.00	.08*	.07*	−.11^**^	−.10^**^	−.07*	.02	−.33^***^	−.29^***^	−.17	.16^***^	−.19^***^	1.00		
**Age (Z2)**	−.06	−.10^**^	−.05	−.05	.02	−.03	.02	.14^***^	−.16^***^	−.01	−.07*	−.09^**^	−.13^***^	1.00	
**Education (Z3)**	−.08*	−.05	.11	.00	.10^**^	.02	−.03	.07*	−.04	−.03	.11^**^	−.04	.00	.01	1.00

*Note.* Values are Person correlation coefficients estimated with SPSS.

* *p*<.05, ** *p*<.01, *** *p*<.001 (two-tailed).

1 Reference group: Male.

**Table 5:** Results of the Four Multiple Linear Regression Analyses to Predict (1) Suicidal Ideation, (2) Depression, (3) Life Satisfaction, and (4) Psychoticism in Study Participants (n = 943).

Predictors and covariates		Suicidal ideation	Depression	Life satisfaction	Psycho-ticism
No. of suicide films (total)	β	−0.02	0.04	−0.09	0.00
	*SE*	0.09	0.09	0.09	0.09
No. of suicide films (%)	β	−0.02	0.01	0.01	0.02
	*SE*	0.05	0.05	0.05	0.05
Suicide film rating	β	0.01	0.08	0.13*	0.11
	*SE*	0.06	0.06	0.06	0.06
Genre: Film noir, Milieu drama	β	0.18^***^	0.11^**^	−0.14^***^	0.20^***^
	*SE*	0.04	0.04	0.04	0.04
Genre: Fantasy, Sci-Fi	β	−0.04	−0.04	0.03	0.05
	*SE*	0.05	0.05	0.05	0.05
Genre: Adventure, Western	β	−0.03	−0.05	0.00	−0.12^**^
	*SE*	0.04	0.04	0.04	0.04
Genre: Tragicomedy, Tragedy, Melodrama	β	0.05	0.11^**^	−0.01	0.04
	*SE*	0.04	0.04	0.04	0.04
Genre: Thriller, Horror	β	0.00	−0.06	−0.01	−0.07
	*SE*	0.04	0.04	0.04	0.04
Sex^1^	β	0.02	0.06	0.03	−0.06
	*SE*	0.04	0.04	0.04	0.04
Age	β	−0.08*	−0.11^**^	−0.02	−0.13^***^
	*SE*	0.04	0.04	0.04	0.04
Education	β	−0.10^**^	−0.08*	0.13^***^	−0.01
	*SE*	0.04	0.03	0.03	0.03

*Note*. Values are standardized regression coefficients (β) and standard errors of the beta coefficients (*SE*) estimated with SPSS.

* *p*<.05, ** *p*<.01, *** *p*<.001 (two-tailed).

1 Reference group: Male.

Documentaries, which were originally not included in our list of film genres due to their non-fictional character (see Table 2), was the most often named film genre preference that the participants of our study felt was missing in our online survey (*n* = 37, 3.9%). However, preferences for documentaries were not associated with suicidal ideation (*F*(4, 943) = 2.79, Adj. *R^2^* = .01, β = 0.04, SE = 0.03, *t* = 1.31, *p* = .19), depression (*F*(4, 943) = 4.33, Adj. *R^2^* = .01, β = 0.03, SE = 0.03, *t* = 0.96, *p* = .34), life-satisfaction (*F*(4, 943) = 4.35, Adj. *R^2^* = .01, β = 0.00, SE = 0.03, *t* = 0.13, *p* = .89), or psychoticism (*F*(4, 943) = 4.57, Adj. *R^2^* = .02, β = 0.06, SE = 0.03, *t* = 1.70, *p* = .09), and was not a significant predictor for classification into suicidal and non-suicidal individuals via Beck Hopelessness Scale (*χ^2^* (4, 943) = 4.47, *p* = .35, Nagelkerkes *R^2^* = .01, β = 0.05, SE = 0.10, *p* = .58, CI = 0.873 to 1.275).

## Discussion

Preferences for certain film genres were significantly associated with risk factors of suicidal behavior, even when controlling for participants' age, gender and education. The more the participants had preferences for film noir movies and milieu dramas, the greater was their suicidal ideation, depression, and psychoticism, and the smaller was their life satisfaction. Furthermore, individuals with preferences for tragicomedies, tragedies and melodramas had significantly higher depression scores. Suicidal individuals, as indicated by high scores on the Beck Hopelessness Scale, also had significantly higher preferences for thrillers and horror films than non-suicidal individuals. These findings are not surprising, since most of these genres provide movies that typically display violence, tragic events and sad environments [35], [36]. Previous studies have acknowledged the link between suicidality and exposure to drama films in general [3], [17], but this is the first time a relationship with specific subcategories of this genre was demonstrated. In contrast, individuals with preferences for adventure movies, westerns, comedies and romantic movies (factor 3) had lower scores for psychoticism, which constitutes another risk factor for suicide. Because comedies and romantic movies focus primarily on the pleasant things in life and tend to display a kind, jolly, and benevolent portrayal of the world [37], this finding suggests that the preference for film genres with happy contents was partially associated with lower scores on suicide risk factors, although not directly with suicidal ideation. The scoring on risk factors of suicide seems to be reflected in the individual film genre preferences suggesting that more vulnerable individuals tend to watch sad films.

Cultivation theory [6] suggests that cumulative exposure to sad and disturbing film contents has the potential to increase or maintain suicidal ideation among viewers, while happy, jolly media contents could help maintain a positive emotional state. Evidence of films impacting on harmful behaviors has been gained with other health-related outcomes, such as smoking. Several studies demonstrated that cumulative exposure to smoking behavior in films increased smoking behavior of the audience [4], [21]–[23]. Watching movies with sad contents may be used to enhance or maintain the current emotional state [36], [38], which may involve the maintaining of suicidal ideation. This interpretation is consistent with Ringel's [39] concept of suicidal constriction, which suggests a drive to solely focus on tragic and sad aspects of life and an increasing tendency to focus on death and suicide in particular. A recent study further demonstrated, that the greater an individual's suicidality was, the more he or she used dramas with sad endings to gather ideas about how to go through life [15], and this approach toward fictional media has been discussed to amplify negative effects of suicide films such as an increase in depression [40]. Thus, it is plausible to assume that individuals with greater suicidal tendencies prefer sad films, because these films reflect their current psychological state, and due to the consumption of sad films they may regulate, maintain or, in the worst case, even increase their suicidality. More research is necessary to investigate psychological mechanisms behind film preferences in vulnerable individuals.

The preference for thrillers and horror movies among suicidal individuals found in the present study is particularly noteworthy, since previous studies neglected the association between fanship of horror movies and suicidality based on the notion that viewers are not able to identify with the unrealistic settings typically portrayed in horror movies [3], [17]. However, the present study shows that suicidal individuals tend to watch horror movies. A potential explanation for this association may be the high amount of violence contained by horror movies. According to the interpersonal theory of suicide by Joiner [41], one way to potentially acquire the capability for serious self-injury and suicide is involvement in violence. Exposure to violent movies and desensitization towards violence could be a component of this process. Correspondingly, a recent study [42] has identified violence, including fighting and weapon carrying, as an appropriate variable to differentiate between suicide ideators and attempters. The results of the present study warrant more research on the link between suicidality and the preference for horror movies.

Exposure to suicide films was not significantly associated with any of the suicide risk factors. This result is surprising, since cumulative exposure to suicide in movies was found to increase the risk of a suicide attempt by 47% in a previous investigation [17]. Bivariate correlations demonstrated a significant association between the number of known suicide films and psychoticism. However, inspection of the correlation and multivariate regression coefficients indicated that this relationship may be mediated by the preference for film noir movies and milieu dramas.

In spite of this pattern of film preferences, the present study also highlights that individual enjoyment of suicide films as indicated by more positive film ratings was generally associated with higher life satisfaction, which is considered a protective factor in suicide. It seems that individuals with higher scores on risk factors for suicide have a tendency to prefer films with sad content, but at the same time they do not experience enjoyment during films with suicide portrayals. In line with social comparison theory [11], exposure to the hopeless situations displayed in suicide films may promote positive self-evaluation, especially among viewers with low or no suicidal tendencies [12]. This may explain the link between positive ratings of suicide films and high life satisfaction. Individuals with low life satisfaction may conversely be less inclined to enjoy such contents.

This study has some limitations. First, the study participants were not representative of the total population, with an overrepresentation of female and young individuals who had finished secondary or higher education. Furthermore, our list of film genres and suicide films was not exhaustive. Individuals may have known other suicide films or preferred other film genres that were not listed on our survey. Further, the reliability of the psychoticism scale was relatively low (see Table 3), which is a known psychometric limitation of the administered instrument [43]. Thus, the results observed with regard to psychoticism should be interpreted with caution. Finally, due to the cross-sectional design of the study it cannot be assessed if film genre preference was causally related to the suicide risk factors.

## Conclusions

Considering the effects of movies presented in many studies in recent decades which report an increase in rates of suicide and attempted suicide due to the broadcasting of films with suicide portrayals [7]–[9] or an increase of symptoms of depression after exposure to drama films [12], [40], the inter-relatedness of personal risk factors for suicide and consumption of media products with suicidal contents warrants a focus in mental health research. The present research is, so far, the largest study that focused on associations between film preferences and risk factors for suicide. Suicidal individuals seem to choose sad films that portray the world in a depressing or disturbing way, which may contribute to the maintenance or increase of suicide risk factors. Future research may seek to explore the specific motives for the consumption of both sad and joyful movies and the emotional and cognitive processing of such films and their impact and perceived meaning, risks and usefulness for vulnerable individuals. Future studies may also seek to investigate the association between suicidality and preferences for other types of entertainment such as music or online contents.

The results of the present study have important implications for suicide prevention. With film genre preferences, in particular the preference for film noir movies and milieu dramas, being associated with risk factors for suicide, it may turn out to be useful to pay more attention to an individual's film consumption, when screening for suicide risk. The preference for this film genre could be added to suicide risk inventories and questionnaires and may help clinicians identifying individuals most at risk for suicide. Further studies, including studies in clinical settings seem warranted to investigate this question further. Clinicians need to be concerned with the media usage history and preferences of their patients and may advise suicidal patients to avoid potentially harmful media contents.
